# Iron Sequestration by Galloyl–Silane Nano Coatings Inhibits Biofilm Formation of *Sulfitobacter* sp.

**DOI:** 10.3390/biomimetics8010079

**Published:** 2023-02-12

**Authors:** Reid E. Messersmith, F. Connor Sage, James K. Johnson, Spencer A. Langevin, Ellen R. Forsyth, Meaghan T. Hart, Christopher M. Hoffman

**Affiliations:** 1 Research and Exploratory Development Department, The Johns Hopkins University Applied Physics Laboratory, 11100 Johns Hopkins Road, Laurel, MD 20723, USA; 2 Asymmetric Operations Sector, The Johns Hopkins University Applied Physics Laboratory, 11100 Johns Hopkins Road, Laurel, MD 20723, USA

**Keywords:** gallic acid, biofilms, antifouling, silanes

## Abstract

Microbially-induced corrosion is the acceleration of corrosion induced by bacterial biofilms. The bacteria in the biofilms oxidize metals on the surface, especially evident with iron, to drive metabolic activity and reduce inorganic species such as nitrates and sulfates. Coatings that prevent the formation of these corrosion-inducing biofilms significantly increase the service life of submerged materials and significantly decrease maintenance costs. One species in particular, a member of the Roseobacter clade, *Sulfitobacter* sp., has demonstrated iron-dependent biofilm formation in marine environments. We have found that compounds that contain the galloyl moiety can prevent *Sulfitobacter* sp. biofilm formation by sequestering iron, thus making a surface unappealing for bacteria. Herein, we have fabricated surfaces with exposed galloyl groups to test the effectiveness of nutrient reduction in iron-rich media as a non-toxic method to reduce biofilm formation.

## 1. Introduction

Microbial induced corrosion is the degradation of metallic surfaces by biofilm-forming microbes. This type of corrosion is responsible for an estimated 20% of the global damages caused by corrosion, amounting to USD 30–50 billion per year in the United States [1]. These biofilms can accelerate localized corrosion of steel by 1000–10,000 fold compared to chemical corrosion alone and are a common cause of “pitting” corrosion [2]. The increased corrosion rate is the result of an increased electrical conductivity in the corrosion zones. The marine environment has nanomolar concentrations of iron [3,4] and most bacteria require micromolar levels for growth, so steel structures are great potential nutrient sources [5]. Microbial biofilms excrete small molecules or grow conductive appendages that rapidly shuttle electrons from the metal surface into the surrounding biofilm. Some microbes secrete flavins and phenazines as electron shuttles, while others grow pili or “nano-wires” towards the metal surface to maintain contact [6]. Through these electron shuttles, microbes in biofilms can readily oxidize metallic iron, using those electrons to drive metabolic function. There are two iron-oxidizing metabolism pathways of interest that differ in energy sources and reduction substrates. Microbes such as *Pseudomonas aeruginosa* and *Shewanella* sp. reduce sulfate or nitrate using electrons as their sole energy source to corrode surfaces, but require equal amounts of reduction substrates [6]. Others, such as *Rhodobacter ferrooxidans* and *Rhodopseudomonas palustris,* do not require fixed electron sinks but utilize sunlight and photosynthesis to energize electrons to reduce CO_2_ [7,8]. The conditions within biofilms enhance microbial-induced corrosion by protecting the bacteria from the external environment, while also serving as a conductive matrix once the microbes excrete electron shuttles or nanowires [9].

Bacteria express siderophores to extract iron from the surrounding environment and this process is closely tied to biofilm formation [10]. Siderophores are small molecules with high binding affinities for iron that also play a role in interspecies communication [11]. There are a large number of marine siderophores [12], and the major categories are catecholates, hydroxamates, and carboxylates. The amount of expression of different types of siderophores has been shown [3] to be dependent on the size and composition of the microbial community. Quorum sensing molecules, including *N*-acylhomoserine lactones, have also been shown in *Pseudomonas aeruginosa* to act as siderophores to bind this critical metal [13]. In *Vibrio harveyi*, the same gene clusters control both siderophore- and quorum-sensing production to the benefit of the bacterial community [10]. The intertwined relationship between the iron-binding siderophores, quorum sensing signaling pathways, and biofilm formation suggests iron-sequestration as a promising method towards reduced microbial induced corrosion.

The *Roseobacter* clade is one of the most widely distributed bacterial groups in the marine ecosystem and is known to form biofilms. It is estimated that this clade constitutes roughly 30% of the bacterial community in open-ocean marine ecosystems [14], and up to 20% in coastal waters [15]. Genomic analyses to date have revealed that the evolutionary success of this clade is at least partially due to a diverse range of regulatory circuits, secondary metabolite production, and trophic strategies (e.g., heterotrophy, photoheterotrophy, or autotrophy) present within the clade [15]. Owing to this prescribed ubiquity and diversity, it is unsurprising that the *Roseobacter* clade is also recognized as the primary colonizer of various organic and inorganic surfaces [16]. A subset of the *Roseobacter* species plays an important biogeochemical role by transforming reduced sulfur compounds, and they have a highly conserved sequence for quorum sensing across 43 different genomes [17,18]. *Sulfitobacter* sp. EE-36 belongs to the culturable subset of *Roseobacter* and was isolated from salt marshes on the coast of Georgia, USA [19]. *Sulfitobacter* sp. EE-36 exhibits high sulfur oxidation activity and is an aggressive surface colonizer that forms biofilms [18,20]. Recently, Tsukidate et al. investigated the promotion of biofilm growth on materials containing high amounts of iron and found *Sulfitobacter* sp. colonized the surface and formed a biofilm [21]. This suggests that *Sulfitobacter* sp. may be more likely to colonize an iron-based ship hull in iron-limiting environments, thus serving as an excellent candidate to test iron-sequestration as a biofilm-preventing measure.

**Figure 1:**
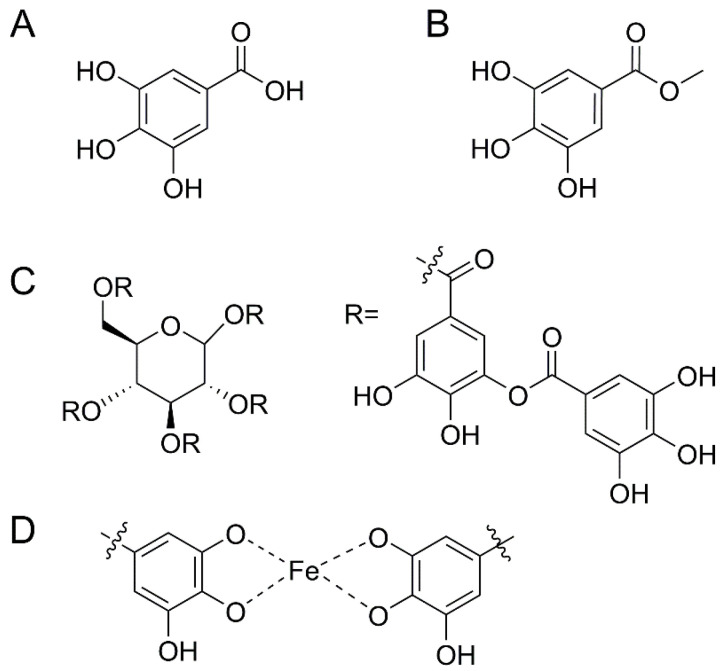
Chemical structures of (**A**) gallic acid, (**B**) methyl gallate, (**C**) tannic acid, and (**D**) galloyl binding of iron.

Galloyl-based chemical moieties are also effective at binding iron [22,23], and iron-limiting strategies have been shown to be effective against biofilm formation [5]. Lactoferrin has been previously demonstrated [24] to limit bacterial growth by sequestering iron from the environment. Iron coordination occurs at marine pH levels through a 2:1 galloyl:iron ratio [25] (Figure 1D) and has a higher binding constant than catechol-bearing molecules [26]. There is a precedent in the literature for catechol antifouling coatings [27], and herein we chose to investigate the galloyl functional group as we hypothesize that the higher binding constant of iron should increase its ability to inhibit biofilm formation. We tested these three galloyl-containing molecules (Figure 1) against *Sulfitobacter* sp. to evaluate their ability to inhibit biofilm formation and then fabricated a monolayer coating of galloyl–silane on glass slides that produced non-toxic and anti-biofilm forming behavior of a biofilm-forming species known to induce microbial induced corrosion. We have not found any previous precedent in the literature for galloyl-functionalized nano coatings and pursued this topic to evaluate the potential efficacy as an antifouling coating.

## 2. Materials and Methods

### 2.1. Bacterial Strain and Culture Conditions

*Sulfitobacter* sp. EE-36 is a gram-negative marine strain that has been isolated from the coastal waters of the southeast United States. *Sulfitobacter* sp. was cultured on marine agar (BD Difco 2216) for 48 h at 26°C. Liquid cultures were prepared by transferring a single plate colony to 5 mL of marine broth (BD Difco 2216). Stationary phase (2.2 OD_600_, 10^8^ CFU mL^−1^) was reached after incubation for 22–24 h at 26 °C with shaking at 120 rpm.

### 2.2. Reagent and Media Preparations

Marine agar was prepared by adding 15 g/L of granulated agar to marine broth mix. Formaldehyde was used to fix bacterial cultures and biofilm structure. A 4% formaldehyde solution was prepared by diluting 37% formaldehyde with phosphate buffer. Crystal violet stain was used to stain bacterial cell walls and biofilm structure. A 1% crystal violet aqueous solution was diluted 1:10 in phosphate-buffered saline to create a 0.1% V/V staining solution. Congo red stain was also used for staining. Congo red stain (pH 7.4) was prepared just before use by adding Congo red (10 mg/mL) to the salt buffer (0.15 M NaCl, 5 mM KH2PO4).

### 2.3. Glass Functionalization

Glass slides were treated in an oxygen plasma cleaner for 15 min.

Solutions of 3-aminopropyl trimethoxysilane (APTMS) were prepared accordingly:

0%: 0 g APTMS, 99 g 95% ethanol, 0.1 g acetic acid;

1%: 1 g APTMS, 99 g 95% ethanol, 0.1 g acetic acid;

5%: 5 g APTMS, 95 g 95% ethanol, 0.1 g acetic acid;

10%: 10 g APTMS, 90 g 95% ethanol, 0.1 g acetic acid;

The plasma cleaned slides were then submerged in the 3-APTMS solutions for 24 h, removed, and cleaned with ethanol followed by DI water.

A solution of gallic acid (0.200 g, 1.18 mmol), HOBT (0.208 g, 1.53 mmol) in DMF (12 mL) was treated with EDC-HCl (0.292 g, 1.53 mmol) and allowed to stir for 1 h. The resulting solution was added to the silane-treated slides and allowed to stand on the slides for 24 h. Following the reaction, the glass slides were washed with ethanol and water to remove excess reagents and then allowed to dry.

### 2.4. Biofilm Formation

Biofilms were developed in 24-well plate culture dishes on the surface of a glass cover slide. Glass cover slides were coated as described above. All cover slides were UV-sterilized (UV-C) on both sides for 45 min before experimentation. One cover slide was placed on the well of the culture dish. The diameter of the cover slide was roughly equivalent to the diameter of the well, such that the cover slide covered the entirety of the flat well bottom.

Cover slides were washed once with distilled water. This step was incorporated to wash away any unwanted chemical residue and aid in sealing the cover slide to the well bottom, which prevented slides from floating during the incubation step. Sterile marine broth or stationary phase *Sulfitobacter* sp. EE-36 cells (6 × 10^6^ CFU/mL, 500 µL) were added to each well. Culture dishes were sealed with a gas-permeable membrane before incubation for 24 h with shaking (120 rpm) at 26 °C.

### 2.5. Cell Suspension Viability

After incubation, the cell suspension was removed for dilution and plating. Sterile suspensions were plated directly onto marine agar. Cell suspensions were diluted to 10^−5^ and 10^−6^ before plating onto marine agar. All plates were incubated for 48–72 h at 26 °C before counting.

### 2.6. Biofilm Imaging

Glass cover slides were gently washed three times with PBS, then 4% formaldehyde (500 µL) was added to each well. The slides were chemically fixed at room temperature for a minimum of one hour. At the end of the fixation period, formaldehyde was removed, and the slides were gently washed three times with distilled water.

In initial screening experiments, crystal violet (0.1%, 300 µL) staining solution was added to each cover slide and allowed to incubate for 20 min. In subsequent experiments, Congo red (10 mg/mL, 500 µL) staining solution was added to each cover slide and incubated for 30 min. The remaining procedure was identical, independent of the stain used. The staining solution was removed from each well, and the slides were gently washed with distilled water one time. Slides were then carefully removed from their wells. Each slide was gently submerged in distilled water, followed by air-drying and placement into a new 24-well culture dish.

Absorbance or fluorescent intensity were measured for each cover slide from staining with crystal violet or Congo red, respectively. Both measurements were made using a fluorescent plate reader (Tecan Infinite M1000). For fluoresce measurements, excitation and emission wavelengths were set to 497 nm 614 nm, respectively. Further analysis was performed via visual inspection under a fluorescent microscope using a TRITC filter.

## 3. Results and Discussion

**Figure 2:**
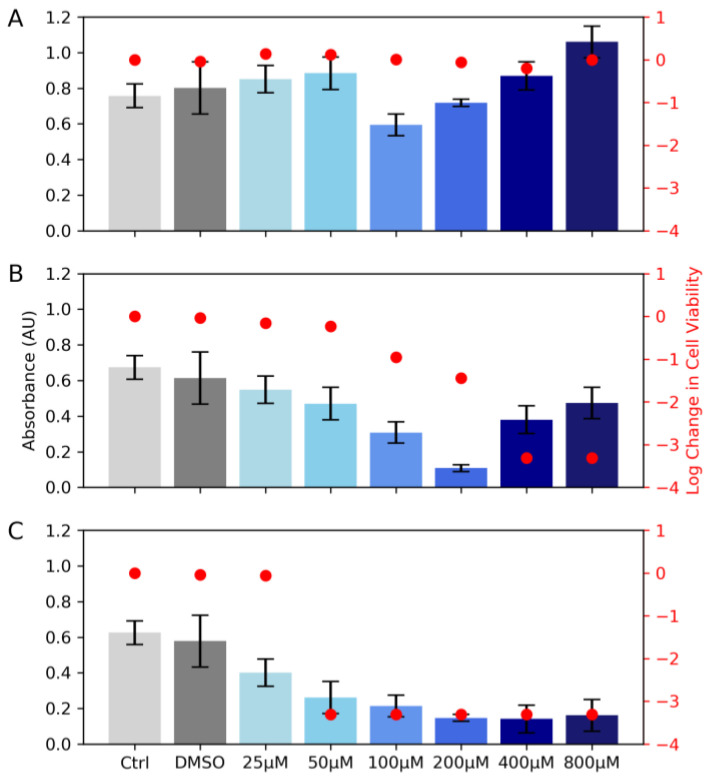
Concentration evaluation of gallic acid (**A**), methyl gallate (**B**), and tannic acid (**C**) with absorbance (left—gray/blue) indicating biofilm formation and log change in cell viability (right—red) reporting the toxicity of the small molecule.

Three molecules: gallic acid, methyl gallate, and tannic acid that contain galloyl functional groups were selected for a concentration-dependent analysis, and the results are shown in Figure 2. Quantification of biofilm formation was performed using crystal violet absorption (Figure 2, left axis), and a cell viability assay was used to determine the toxicity of the coating (Figure 2, right axis). Compared to the water or DMSO controls, an increase in gallic acid from 25 μM to 800 µM did not show a clear trend with biofilm formation but did maintain a lack of toxicity (Figure 2A). Methyl gallate decreased the biofilm formation between 25 μM to 200 µM, but there are issues with toxicity above that concentration (Figure 2B). Tannic acid inhibited the biofilm growth but likely in a toxic pathway, as shown with the Log Change data from the cell viability assay (Figure 2C). This may also be relative to the amount of galloyl groups, as a 25 µM solution of tannic acid has four more galloyl groups than gallic acid at the same concentration. The goal is a coating that decreases biofilm formation but is non-toxic, so the behavior of methyl gallate between 25 μM to 200 µM was found to be preferable. Additionally, the comparison between gallic acid and methyl gallate was interesting because the data in Figure 2 show that the free carboxylic acid in gallic acid does not lead to a beneficial result. This suggests that as we move from dissolved small molecule tests to surface-bound tests, the covalent bond between the galloyl group and the surface should not inhibit but improve the biofilm growth inhibition.

**Figure 3:**
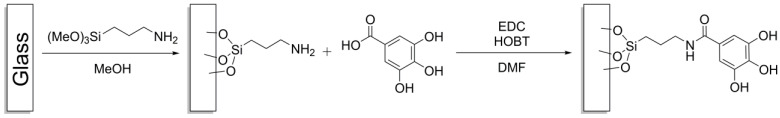
Two-step surface functionalization of (1) silanization followed by (2) amidation coupling.

The dissolved small molecule experiment results suggest that the galloyl portion of the molecule is relevant for inhibiting biofilm growth. Galloyl groups were covalently tethered to the surface, with the aromatic alcohols exposed to the sequester iron to create a surface that prevents biofilm formation. The galloyl monolayer coating was fabricated via a two-step process: (1) the functionalization of the glass with 3-aminopropyl trimethoxysilane (APTMS) and then (2) the coupling of gallic acid to the pendant amine groups to form a gallate-silane (Figure 3). As the concentration was a relevant factor for performance, we fabricated samples with 0, 1, 5, and 10% silane loadings and then an excess of gallic acid in appropriate coupling conditions to generate the final coating. All coatings were exposed to identical bacterial conditions and treated equally so that the only difference between the samples was the galloyl loading.

**Figure 4:**
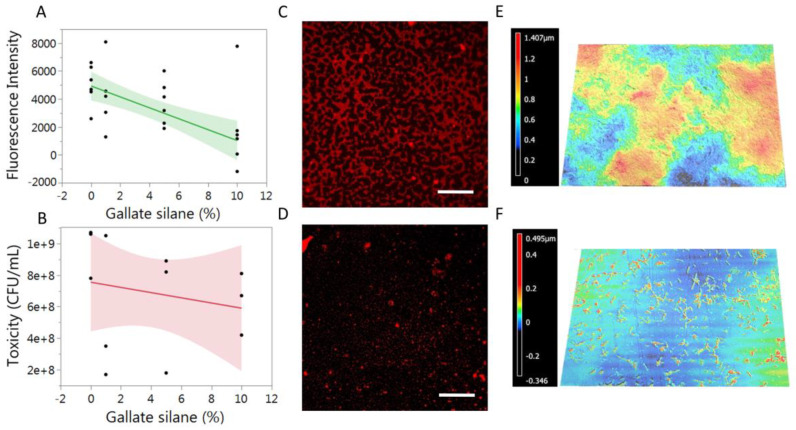
Fluorescence was measured from stained *Sulfitobacter* sp. biofilms on glass treated with (**A**) various concentrations of gallate–silane and (**B**) bacteria concentration (CFU/mL) of the inoculating culture after 24-h exposure to the silane coatings. Fluorescent microscope images (**C**,**D**) and Keyence height profiles (**E**,**F**) of glass slides with (**top**) 0% gallate–silane and (**bottom**) 5% gallate-silane (scale bar 50 µM).

Functionalized glass slides were then exposed to marine broth inoculated with *Sulfitobacter* sp. and allowed to incubate for 24 h. The resulting biofilm was then stained using Congo Red dye, which can preferentially stain the carbohydrate components of the extracellular matrix of biofilms, and the fluorescence was measured. As the concentration of gallate–silane on the surface was increased, the resulting fluorescence decreased, suggesting that there was significantly less biofilm growth on the slide (Figure 4A). Additionally, the enriched marine broth was plated, and any bacteria still living in the culture were allowed to grow. It was found that there was no statistically significant decrease in the concentration of bacteria in the culture, suggesting that the coatings that were applied were non-toxic (Figure 4B). Fluorescent microscope images of the samples confirmed the findings from the fluorescent assay. Figure 4C shows an interconnected biofilm on the 0% gallate–silane but no obvious biofilm on the 5% gallate–silane-treated substrates, as in Figure 4D. Rather, individual cells and small clusters were apparent. Since *Sulfitobacter* sp. is known to seek out iron when forming biofilms, we propose that the iron sequestration of the nano coating inhibited the growth on these surfaces. This highly modular surface functionalization enables future work on a diverse array of small molecules on readily available glass surfaces.

## 4. Conclusions

We demonstrated that the galloyl motif has concentration-dependent action against *Sulfitobacter* sp. There is a significant decrease in biofilm formation with an increasing concentration of gallate–silane in the monomer layer. The minimal response in biofilm formation to gallic acid, as opposed to the reduction with methyl gallate, highlights that the carboxylic acid is not a key functional group effector. Thus, we can make a gallate–silane monolayer that has exposed alcohol groups to impact the biofilm growth. The lack of toxicity from the gallate–silane studies suggests that the mode of action is local nutrient depletion via iron sequestration. Future work will focus on more complex testing scenarios as galloyl-based nano coatings are a promising route for antifouling surface coatings with non-toxic functionality.
